# The dataset of bat (Mammalia, Chiroptera) occurrences in Ukraine collected by the Ukrainian Bat Rehabilitation Center (2011-2022)

**DOI:** 10.3897/BDJ.11.e99243

**Published:** 2023-03-07

**Authors:** Alona Prylutska, Maryna Yerofeieva, Valeria Bohodist, Alona Shulenko, Anzhela But, Ksenia Kravchenko, Oleh Prylutskyi, Anton Vlaschenko

**Affiliations:** 1 Ukrainian Bat Rehabilitation Center, Ukrainian Independent Ecology Institute, Kharkiv, Ukraine Ukrainian Bat Rehabilitation Center, Ukrainian Independent Ecology Institute Kharkiv Ukraine; 2 Max-Planck Institute of Animal Behaviour, Department of Migration, Radolfzell, Germany Max-Planck Institute of Animal Behaviour, Department of Migration Radolfzell Germany; 3 V. N. Karazin Kharkiv National University, Kharkiv, Ukraine V. N. Karazin Kharkiv National University Kharkiv Ukraine; 4 Bila Tserkva National Agrarian University, Bila Tserkva, Ukraine Bila Tserkva National Agrarian University Bila Tserkva Ukraine; 5 H.S. Skovoroda Kharkiv National Pedagogical University, Kharkiv, Ukraine H.S. Skovoroda Kharkiv National Pedagogical University Kharkiv Ukraine; 6 University of Lausanne, Department of Ecology and Evolution, Lausanne, Switzerland University of Lausanne, Department of Ecology and Evolution Lausanne Switzerland

**Keywords:** Chiroptera, *Nyctalusnoctula*, *Eptesicusserotinus*, *Pipistrelluskuhlii*, *Vespertiliomurinus*, Vespertilionidae bats, Kharkiv City, urban-landscapes

## Abstract

**Background:**

Bats are of high conservational status in most European countries. All bats are under legal protection in Ukraine and included in the national Red Data Book. However, bats remain one of the least studied groups of mammals in Ukraine. Their cryptic lifestyle limits the possibilities of direct observations and, as a result, data on bat distribution are incomplete. Wildlife rehabilitation centres accumulate a plethora of records of wild animals and those data may significantly contribute to knowledge on the species range, phenology and habitat preferences.

This paper presents the data accumulated from over a decade of work by the Ukrainian Bat Rehabilitation Center (formerly The Bat Rehabilitation Center of Feldman Ecopark), the premier organisation engaged in the rescue and rehabilitation of bats across Ukraine. In addition to in-person data collected by Ukrainian Bat Rehabilitation Center staff, the Center also accepts observations of bat encounters from citizens. The Center’s dataset boasts over 20,000 distinct observations, which are the subject of this paper.

**New information:**

This dataset, spanning 2011-2022, contains a total of 20,948 records of bat findings, 19,024 of which consist of records directly identified by UBRC team members. The remaining 1924 observations were provided by citizens through helpline. Data on 16 species and one subspecies have been collected. The highest number of records belongs to *Nyctalusnoctula* (n = 15889), followed by *Eptesicusserotinus* (n = 2017) and *Pipistrelluskuhliilepidus* (n = 2001). Less than 10% of these records have been previously published; the rest are presented in this paper for the first time. The dataset is particularly rich in information on bats in human settlements and is (to the best of the authors’ knowledge) the largest dataset on bats within human-modified landscapes ever collected from the territory of Eastern Europe. The entire dataset is available through the Global Biodiversity Information Facility (GBIF).

## Introduction

Amongst the list of highly prioritised for mammal conservation, bats are taking a very distinctive place globally. European bats, in particular, have been recognised as key bioindicators of habitat quality and land-use change (Jones et al. 2009, Russo and Jones 2015). Bat roosting sites and feeding habitats are protected by European Union and national laws in almost every country in Western and Central Europe. In Ukraine, all bat species are included in the national Red Data Book and legally subject to stringent environmental protections. The keystone for establishing animal and habitat conservation programmes are datasets of species distribution collected by professional researchers, volunteers, as well as both Government and Non-Governmental organisations (Akimov 2009). While bats are subject to legal protection in Ukraine, the quantity and quality of data on their distribution lag behind those available for Western and Central Europe, which significantly impedes the development and implementation of conservation efforts at the national scale.

Despite the much lower species diversity in Europe than in the tropics, precise bat species identification remains challenging. In the 20^th^ century, such identification and data collection relied chiefly on the efforts of professional zoologists with specific skills, experience and knowledge (e.g. Strelkov and Il'in (1990)). In Ukraine, the data about bat distribution mainly were collected in natural habitats during dedicated zoological expeditions though encounters between citizens and bats were unlikely less common than they are today. However, awareness of the importance of zoological data, lack of direct information channels to experts and the expense of making reliable records (photos or videos) limited the potential for any sort of data crowd-sourcing. The recent spread of the Internet and the availability of high-quality video and photo equipment in smartphones have drastically changed the situation. Additionally, access to the professional identification keys was simplified and the possibility of taking immediate pictures of an animal from different perspectives and passing these pictures to an expert appeared. The websites of local bat conservation and research groups have become hubs for collecting information about bat records both locally and nation-wide. Meanwhile, a spatial focus in bat records has shifted from natural habitats to human settlements. In Ukraine, this shift took place about 15 years ago and the first papers analysing bat records obtained through helplines were published ten years ago (Godlevskaya 2012, Prylutska and Vlaschenko 2013). Thus, with modern technology's help, data collection on bat records in the human-modified landscape has been significantly facilitated.

Bat rescue work in Kharkiv City began in 1999 and was carried out on a voluntary basis by local biology students (Vlaschenko 1999). This informal work continued until 2013, when the Ukrainian Bat Rehabilitation Center of Feldman Ecopark (BRC-FE) was formally founded. Since March 2022, the initiative is continuing, working as the Ukrainian Bat Rehabilitation Center (2022) (UBRC) and partnered with the Non-Governmental organisation "Ukrainian Independent Ecology Institute"' as an umbrella organisation for bat rescue and conservation work on a national scale (Vlaschenko et al. 2022). Aside from its rescue/rehabilitation mission, the UBRC also collects data for scientific analysis (phenology, distribution, ecology of bats). Data on bat records fall into two categories: "correspondence" records and "direct" records. Specialists of the UBRC carefully collect all information about bat records made by citizens that are possible to identify by obtained photo and video material. We marked such records in the dataset as "correspondence records". The second data source consists of bats rescued by UBRC specialists or otherwise transported to the Center. All such bats, whether alive or dead, are carefully examined for body conditions and measured by specialists and their details are recorded. We marked these records in the dataset as "direct records". For the first several years of its existence, the BRC-FE was the only bat rehabilitation center in Ukraine and the Center's work produced a greater amount of data than was possible to process at the time. Several papers published over the years have analysed some of the available data by case (Vlaschenko et al. 2019), time period (Kravchenko et al. 2017) and species (Hukov et al. 2020b); however, the main part of the dataset remained unpublished. By publishing this data (Prylutska et al. 2022), we aimed to present to a wide audience: (i) more than 19,024 direct bat records collected from 2013 to 2021 and (ii) 1,924 correspondence records collected between 2011 and 2022 and spanning the entire territory of Ukraine.

## Project description

### Title

Northern Eurasia 2022

## Sampling methods

### Study extent

We collected the direct records for 2013-2021 and correspondence records for 2011-2022 from all the territory of Ukraine.

### Sampling description

The data in this dataset were produced by citizens and specialists of UBRC during bat rescue operations in human settlements. Most records represented accidental encounters with bats found on the ground or inside buildings. Another smaller part of the records describes hibernation bat colonies found in buildings during renovations and window replacement (Kravchenko et al. 2017, Hukov et al. 2020b). These colonies are usually single-species. Almost all alive bats we subsequently banded with special bat aluminium rings (manufactured by Aranea, Poland and available in three sizes) marked as “Kyiv, Ukraine” and a unique number (Vlaschenko et al. 2020). We included all recaptured bats (those already banded) in the total sum of bat records. To save information about each individual in the colony, we decided to keep separate rows for them.

Citizens who found bats were able to contact the UBRC through a telephone helpline or via social media messenger apps. Where possible, found bats were delivered to the UBRC facilities in Kharkiv City (NE Ukraine) for detailed examination and rehabilitation. When bats could not be delivered to the UBRC, species identification was made by video or photo (if it were possible, we attempted to identify the sex of an individual) (Prylutska and Vlaschenko 2013). The present dataset contains two types of records: (i) direct (physical) records consisting of bats that were delivered to the UBRC’s office and examined by specialists (“occurrences” in basiOfRecord term in the dataset); (ii) correspondence (electronic) records, bats that were identified from picture(s), provided by the finder (“human observations” in basiOfRecord term). Groups of bats (colonies) that were recorded as correspondence findings are merged in the dataset in one row, with a number of individuals for each colony mentioned in IndividualCount column. Bats brought in during the warm months (April - October) were released immediately after rehabilitation, those brought in during the cold months were held in hibernation until the spring and then released. All bats with signs of injury were examined and treated by a qualified veterinarian. Bats incapable of flying even after treatment were left at the UBRC for life-long care and rehabilitation. For the details of the UBRC's protocols for bat care, rehabilitation, ringing and release, see Domanska et al. (2017) and Vlaschenko et al. (2020). Bat species were identified by UBRC staff using illustrated keys (Dietz and Kiefer 2014, Dietz and Helversen 2004 and Dietz and Kiefer 2014). The methods for identification of bat age, sex and measurements have already been published in detail by members of the UBRC (Hukov et al. 2020b, Kravchenko et al. 2017, [Bibr B8295029][Bibr B8294913], Prylutska et al. 2021). Briefly, we classified bats by age into one of three categories: (i) juvenile - recently born, incapable of flight (ii) subadult - this-year-born individuals, usually ranging in age from 1 to 10-11 months and (iii) adult (Kravchenko et al. 2017). The exact details of categorisation varied by sex as follows. Females of bats with protuberant nipples (1 mm or more in diameter) and milk-white, abraded canine teeth were classified as adults. Females with flat and pink nipples and pinkish, sharp canine teeth were classed as first-year individuals (Kravchenko et al. 2017, Prylutska et al. 2021). Males with milky-white and worn canine teeth, large testes (from 7 × 4 mm or more) and distended, filled epididymis were classed as adults. Males with pinkish and sharp canine teeth, small testes and small, undescended epididymis were classed as first-year individuals (Kravchenko et al. 2017, Prylutska et al. 2021). For each bat we received or being reported, we recorded an address (region, district, settlement name and, for many cases, also street and building number). Coordinates of centroids for each location were obtained by batch geocoding using Google Maps Geocoding API (https://developers.google.com/maps/documentation/geocoding), implemented in Awesome Tables extension for Google Spreadsheet (https://awesome-table.com/) then manually checked using QGIS 3.22 (QGIS Development Team 2022). Coordinate uncertainty for each centroid (in metres) was assigned, based on the precision of the provided address. For the locations specified up to the street level and coarser, coordinate precisions were calculated as a minimal radius of the circle including the whole location. As a reference, we used Ukraine's official administrative boundaries GIS layers (temporarily unavailable in open access due to wartime restrictions); all geocalculations were performed in QGIS 3.22 (QGIS Development Team 2022). We used the WGS 84 / Pseudo-Mercator coordinate reference system (EPSG:3857) to project the bat records map.

### Quality control

Bats delivered to the UBRC office were examined and measured by qualified specialists (biologists and veterinarians). For each individual, the sex, age category, reproductive status, forearm length (accuracy 0.1 mm) and body mass (accuracy 0.1 g) were recorded. All recaptured bats (those already banded) were included in the total sum of bat records. For bat species identification, we used the key developed by Dietz and Helversen (2004) and the identification by teeth with the loupe was done using key from Schober and Grimmberger (1988). For individuals whose species identification was uncertain, only the genus was recorded. For correspondence records, reporters were asked to provide the required details or pictures/video to enable an exact identification of the species. All cases where there was doubt about the genus identification were excluded from the dataset.

### Step description

1. For bats transferred to the UBRC (direct records): identification of bat species, sex and age, measurement of body mass and forearm length, banding with identification bands. All details and measurements were entered into a Google spreadsheet alongside date and location found.
2. For bats not transferred to the UBRC (correspondence records): identification of bat species by photos or videos. All available details (species, sex) were entered into a Google spreadsheet alongside date and location found.
3. Aggregating direct and correspondence records in Libre Office Calc spreadsheet.
4. Manual georeferencing of records, based on descriptions of the localities using Google Maps (Google 2021)
5. Data post-processing using Darwin Core terms (Wieczorek et al. 2012).
6. Data cleaning using OpenRefine (OpenRefine 2022).
7. Dataset publishing on GBIF https://www.gbif.org/dataset/af0a7284-a634-4082-8c14-ab1f7030775b
8. Visualisation of bat correspondence records on the accumulation map using Carto service https://carto.com.

## Geographic coverage

### Description

Ukraine, all the territory.

### Coordinates

44.402 and 52.483 Latitude; 22.236 and 39.99 Longitude.

## Taxonomic coverage

### Description

**Figure 1.**
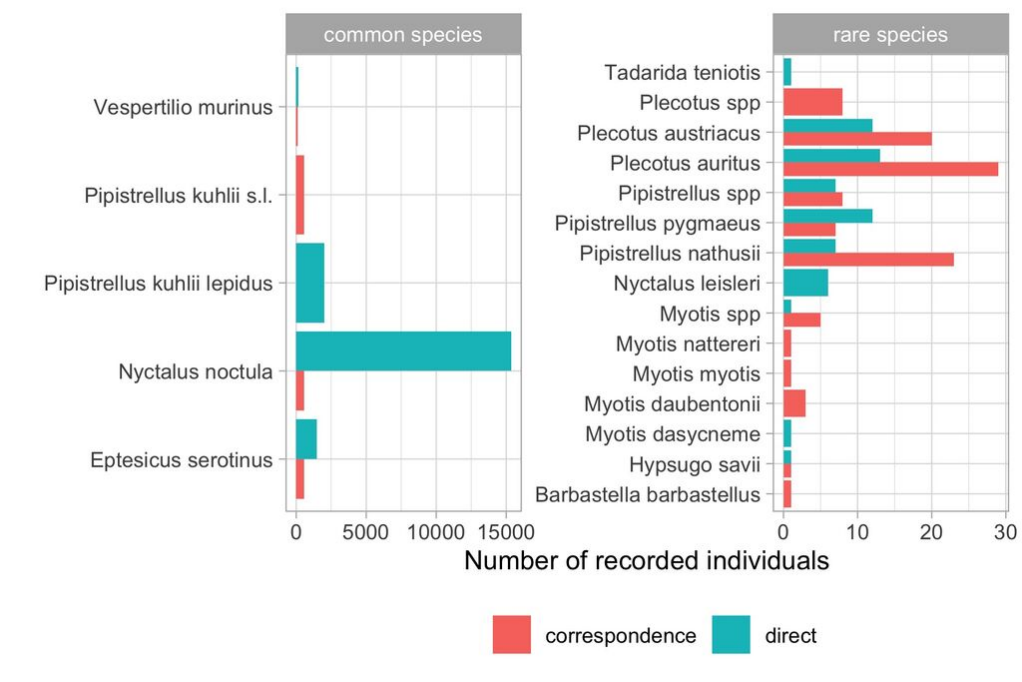
The number of recorded bat individuals collected by the Ukrainian Bat Rehabilitation Center in 2011-2022 (n = 20948). Since there was a huge difference in abundance between species, we plotted "common" (more than 1000 individuals) and "rare" (less than 1000 individuals) species separately. We used these terms for illustrative purposes only and do not imply conservation meaning.

Taxonomic coverage of the dataset consists of Mammals from the order Chiroptera. In total, data on 16 species and one subspecies were collected, which is more than half of the total bat fauna of Ukraine (Akimov 2009). *Nyctalusnoctula* (Schreber, 1774) was the most common species (n = 15889) in our dataset. The least recorded species were three *Myotis* species and *Barbastellabarbastellus* (Schreber, 1774) (n = 1) (Fig. 1). In total, 12 species and one subspecies were recorded by direct records, 11 species from Vespertilionidae belonging to seven genera and one species of bats belongs to the family Molossidae (Prylutska et al. (2020)) (Table 1). By correspondence records, 13 species from the family Vespertilionidae were recorded belonging to eight genera. For most bat species present in the dataset, there were no nomenclatural changes or developments for the last 10 years and their taxonomic status is definite (Dietz and Kiefer 2014). The exception is *Pipistrelluskuhlii* (Kuhl, 1817), presented in Europe by two well-distinguishable visual taxa - *Pipistrelluskuhliikuhlii* (Kuhl, 1817) and *P.k.lepidus* (Andriollo et al. 2015, Sachanowicz et al. 2017). For the whole of the Ukrainian territory, only *Pipistrelluskuhliilepidus* (Blyth, 1845) was identified till now (Sachanowicz et al. 2017, Hukov et al. 2020a). However, there was a noted species range expansion for *P.k.kuhlii* from west to east and it was hypothesised that it would appear in most western regions of Ukraine soon (Sachanowicz et al. 2017). All the bats identified previously (by UBRC specialists) as *P.kuhlii* were later re-evaluated as *P.k.lepidus*. Yet, we cannot confirm whether *P.kuhlii* from the western part of Ukraine (identified by photos), was actually *P.k.lepidus* and not a *P.k.kuhlii*. For this reason, we categorised correspondence records to a higher-level species taxon *P.kuhlii*, but direct records were marked as *P.k.lepidus*.

### Taxa included

**Table:** 

Rank	Scientific Name	Common Name
kingdom	Animalia	Animals
order	Chiroptera	Bats
family	Vespertilionidae	Microbats
family	Molossidae	Free-tailed bats

## Temporal coverage

### Notes

2011 through 2022

## Usage licence

### Usage licence

Creative Commons Public Domain Waiver (CC-Zero)

### IP rights notes

This work is licensed under a Creative Commons Attribution (CC-BY) 4.0 License.

## Data resources

### Data package title

The dataset of bat (Chiroptera, Mammalia) occurrences in Ukraine collected by the Ukrainian Bat Rehabilitation Center (2011-2022)

### Resource link

https://www.gbif.org/dataset/af0a7284-a634-4082-8c14-ab1f7030775b

### Alternative identifiers

https://doi.org/10.15468/7t4zgc

### Number of data sets

1

### Data set 1.

#### Data set name

The dataset of bat (Chiroptera, Mammalia) occurrences in Ukraine collected by the Ukrainian Bat Rehabilitation Center (2011-2022)

#### Data format

Darwin Core

#### Character set

UTF-8

#### Download URL

https://www.gbif.org/dataset/af0a7284-a634-4082-8c14-ab1f7030775b

#### Description

The dataset includes a tabulation-delimited table with 30 fields in Darwin Core terms and 20,948 records. A description of the column headers used is given below.

**Data set 1.** 

Column label	Column description
occurrenceID	https://dwc.tdwg.org/terms/#dwc:occurrenceID; an identifier of a particular occurrence, unique within this dataset. We used a combination of the organisation’s abbreviation, year and incremental numbers.
basisOfRecord	https://dwc.tdwg.org/terms/#dwc:basisOfRecord; the method by which data were acquired. Two levels: “Occurrence" for direct records of bats delivered to the UBRC office and examined by specialists and "HumanObservation" for correspondence records, bats that were identified by the picture(s)/video(s).
eventDate	https://dwc.tdwg.org/terms/#dwc:eventDate; the full date of the observation.
scientificName	https://dwc.tdwg.org/terms/#dwc:scientificName; the original scientific name.
kingdom	http://rs.tdwg.org/dwc/terms/kingdom; the full scientific name of the kingdom in which the taxon is classified.
taxonRank	https://dwc.tdwg.org/terms/#dwc:taxonRank; the taxonomic rank of the most specific name in the scientificName.
identifiedBy	http://rs.tdwg.org/dwc/terms/identifiedBy; a list of names of people who assigned the Taxon to the subject.
locality	http://rs.tdwg.org/dwc/terms/locality; the specific description of the place (in Russian).
decimalLatitude	http://rs.tdwg.org/dwc/terms/decimalLatitude; the geographic latitude in decimal degrees.
decimalLongitude	https://dwc.tdwg.org/terms/#dwc:decimalLongitude; the geographic longitude in decimal degrees.
geodeticDatum	https://dwc.tdwg.org/terms/#dwciri:geodeticDatum; the geodetic datum upon which the geographic coordinates are given. All values are WGS84.
coordinateUncertaintyInMetres	http://rs.tdwg.org/dwc/terms/coordinateUncertaintyInMeters; the horizontal uncertainty distance (in metres) from the given decimal Latitude and decimal Longitude.
georeferencedBy	https://dwc.tdwg.org/terms/#dwc:georeferencedBy; persons who determined the georeference.
georeferencedDate	http://rs.tdwg.org/dwc/terms/georeferencedDate; the date on which the Location was georeferenced.
georeferenceProtocol	https://dwc.tdwg.org/terms/#dwciri:georeferenceProtocol; a description of the method used to determine coordinates.
georeferenceSources	http://rs.tdwg.org/dwc/iri/georeferenceSources; a list of maps used to georeference the Location.
continent	http://rs.tdwg.org/dwc/terms/continent; one value – Europe.
country	https://dwc.tdwg.org/terms/#dwc:country; one value – Ukraine.
countryCode	https://dwc.tdwg.org/terms/#dwc:countryCode; one value – UA.
stateProvince	https://dwc.tdwg.org/terms/#dwc:stateProvince; the name of the administrative region of Ukraine in which the Location occurs (name of the administrative region - Oblast or Autonomous Republic of Crimea or Kyiv City).
language	https://dwc.tdwg.org/terms/#dc:language; one value - en | ru, because each observation combined fields both in English and Russian.
organismRemarks	http://rs.tdwg.org/dwc/terms/organismRemarks; notes about the Organism instance: alive, dead, recapture.
recordNumber	http://rs.tdwg.org/dwc/terms/recordNumber; an identifier (special band number) given to the object at the time it was recorded.
recordedBy	https://dwc.tdwg.org/terms/#dwc:recordedBy; a person or group of people who were the primary collector or observer.
individualCount	http://rs.tdwg.org/dwc/terms/individualCount; the number of individuals present at the time of the Occurrence.
organismQuantityType	https://dwc.tdwg.org/terms/#dwciri:organismQuantityType; the type of quantification system used for the quantity of organisms. “individuals” for the most of occurrences, but in some cases also “colony” for groups with no exact information on quantity.
organismQuantity	https://dwc.tdwg.org/terms/#dwc:organismQuantity; a number for the quantity of organisms, according to the values in the organismQuantityType field.
sex	http://rs.tdwg.org/dwc/terms/lifeStage; the age class of the Organism(s) at the time the Occurrence was recorded.
lifestage	http://rs.tdwg.org/dwc/terms/lifeStage; the age class of the Organism(s) at the time the Occurrence was recorded.
occurrenceRemarks	http://rs.tdwg.org/dwc/terms/occurrenceRemarks; notes about the place of Occurrence.

## Additional information

**Figure 2.**
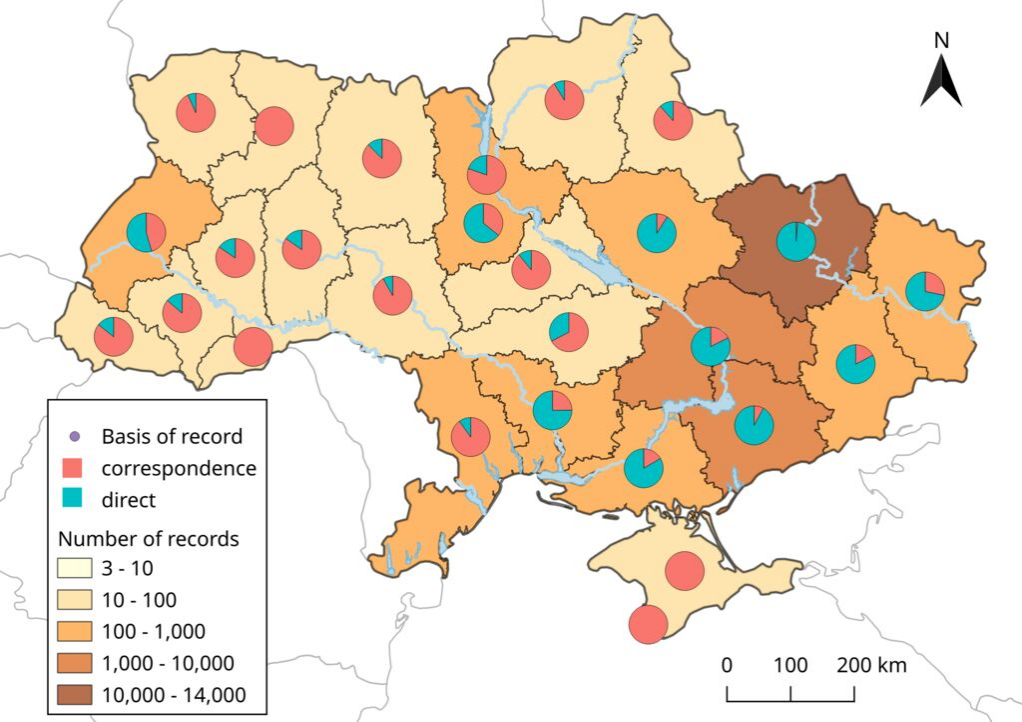
Bat records distribution map of Ukraine (2011-2022). Fill of the Oblast (administrative region) reflects the number of records and pie-charts show the percentage of direct and correspondence type of records.

Amongst all recorded individuals, the most common species was *N.noctula* (Fig. 1). This species is common in the summer-time in Central and North Ukraine (e.g. Gashchak et al. (2013), Vlaschenko et al. (2022a)). For the last twenty-thirty years, *N.noctula* formed numerous winter aggregations in cities of Central and Eastern Europe, including Ukraine (e.g. Godlevska (2015), Kravchenko et al. (2020)). The occurrence of these mass wintering aggregations explains the dominance of this species in our dataset. The following three species in number (*P.k.lepidus*, *E.serotinus* and *V.murinus*) in our dataset are common urban- and rural-dwelling bats in Ukraine (e.g. Vlaschenko et al. (2021)). The rest of the species are mostly dwellers of natural habitats (woodlands, wetlands and rural with a lower level of urbanism) which explains the rarity of their findings in settlements. The distribution of records by administrative regions (Oblast) of Ukraine is presented in Fig. 2. The main records are concentrated in Kharkiv Region and two industrial regions - Dnipropetrovsk and Zaporizhzhia. Generally, more records were made in regions whose capitals are cities with a higher population (Kyiv, L’viv, Odesa, Mykolaiv, Kherson etc.).

**Figure 3.**
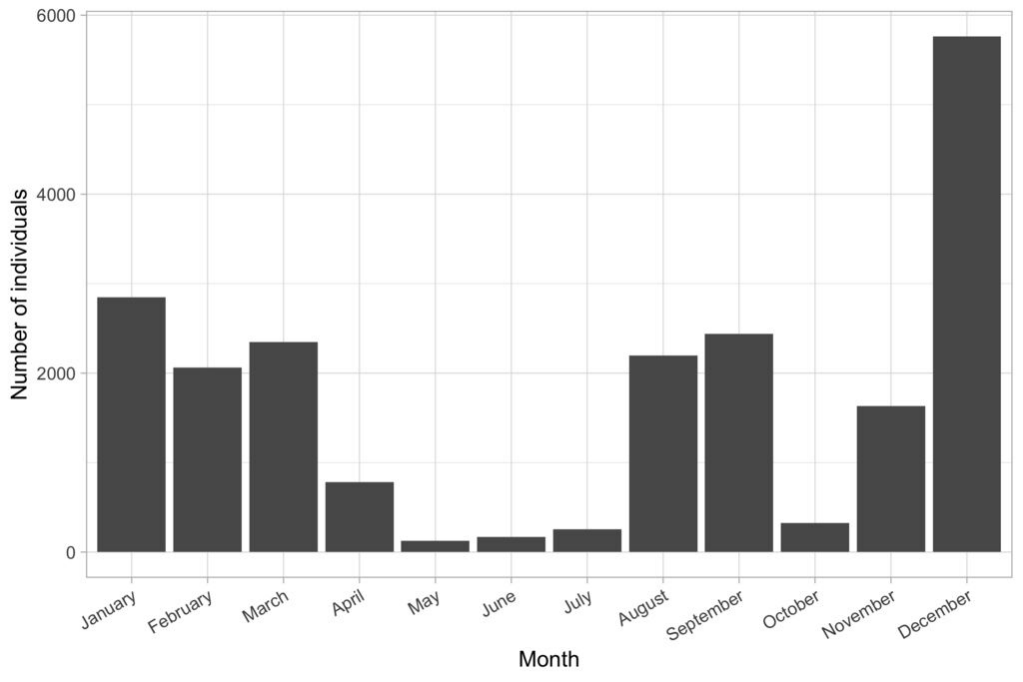
Number of bat individuals collected in different months by the Ukrainian Bat Rehabilitation Center in Ukraine in 2011-2022.

The accumulated number of bats recorded each month during the year is presented in Fig. 3. The number of bats per month represents bats' utilisation of urban and urbanised landscapes and refers to a picture already described for Kharkiv City (Kravchenko et al. 2017). The months with maximum bat records are months with mainly cold weather (November - March), with the second peak in records being August-September (Fig. 3). Thus, the maximum number of bat records appeared in seasons and months when bats do not need a lot of food – hibernation and autumn swarming. On the other hand, during the periods of time when batsrequire the maximum amount of food – breeding season (May - July) and pre-hibernation fat accumulation (October), we obtained the minimum number of records.To show the accumulation of bat correspondence records collected for the dataset, we create the accumulation map video using Carto service (Fig. 4)

## Figures and Tables

**Figure 4.** The accumulation map of bat correspondence records collected by the Bat Rehabilitation Center in Ukraine during the years 2011 - 2022.

**Table 1.** List of the taxonomic coverage of the bats collected as direct and correspondence records by the Ukrainian Bat Rehabilitation Center in Ukraine in 2011-2022.

Class	Order	Family	Genus	Full species name	D.*	C.**
Mammalia	Chiroptera	Vespertilionidae	*Myotis*	*Myotisdaubentonii* (Kuhl, 1817)	-	+
Mammalia	Chiroptera	Vespertilionidae	*Myotis*	*Myotisdasycneme* (Boie, 1825)	+	-
Mammalia	Chiroptera	Vespertilionidae	*Myotis*	*Myotisnattereri* (Kuhl, 1817)	-	+
Mammalia	Chiroptera	Vespertilionidae	*Myotis*	*Myotismyotis* (Borkhausen, 1797)	-	+
Mammalia	Chiroptera	Vespertilionidae	*Nyctalus*	*Nyctalusleisleri* (Kuhl, 1817)	+	-
Mammalia	Chiroptera	Vespertilionidae	*Nyctalus*	*Nyctalusnoctula* (Schreber, 1774)	+	+
Mammalia	Chiroptera	Vespertilionidae	*Eptesicus*	*Eptesicusserotinus* (Schreber, 1774)	+	+
Mammalia	Chiroptera	Vespertilionidae	*Pipistrellus*	*Pipistrelluspygmaeus* (Leach, 1825)	+	+
Mammalia	Chiroptera	Vespertilionidae	*Pipistrellus*	*Pipistrellusnathusii* (Keyserling et Blasius, 1839)	+	+
Mammalia	Chiroptera	Vespertilionidae	*Pipistrellus*	*Pipistrelluskuhlii* (Kuhl, 1817)	+	+
Mammalia	Chiroptera	Vespertilionidae	*Pipistrellus*	*Pipistrelluskuhliilepidus* (Blyth, 1845)	+	-
Mammalia	Chiroptera	Vespertilionidae	*Vespertilio*	*Vespertiliomurinus* (Linnaeus 1758)	+	+
Mammalia	Chiroptera	Vespertilionidae	*Hypsugo*	*Hypsugosavii* (Bonaparte, 1837)	+	+
Mammalia	Chiroptera	Vespertilionidae	*Plecotus*	*Plecotusaustriacus* (Fischer, 1829)	+	+
Mammalia	Chiroptera	Vespertilionidae	*Plecotus*	*Plecotusauritus* (Linnaeus, 1758)	+	+
Mammalia	Chiroptera	Vespertilionidae	*Barbastella*	*Barbastellabarbastellus* (Schreber, 1774)	-	+
Mammalia	Chiroptera	Molossidae	*Tadarida*	*Tadaridateniotis* (Rafinesque, 1814)	+	-

* - direct records; ** - correspondence records.
